# A Novel Medium for Enhancing Callus Growth of Hazel (*Corylus avellana* L.)

**DOI:** 10.1038/s41598-017-15703-z

**Published:** 2017-11-15

**Authors:** Mina Salehi, Ahmad Moieni, Naser Safaie

**Affiliations:** 1 Department of Plant Breeding and Biotechnology, Faculty of Agriculture, Tarbiat Modares University, Tehran, P.O. Box: 14115-336 Iran; 2 Department of Plant Pathology, Faculty of Agriculture, Tarbiat Modares University, Tehran, P.O. Box: 14115-336 Iran

## Abstract

Paclitaxel is a powerful antimitotic agent with excellent activity against a range of cancers. Hazel has been described as a paclitaxel-producing species among angiosperms. Fast-growing callus is a prerequisite for the success of callus production and then paclitaxel production. Therefore, optimizing the medium culture for enhancing callus growth is a crucial step for paclitaxel production. In this research, Murashige and Skoog (1962) (MS) medium was optimized for improving callus growth of hazel (*Corylus avellana* L.). The M_10_ medium (MS medium with pH 6.0 and supplemented with 1000 mg l^−1^ spirulina powder, 1000 mg l^−1^ casein hydrolysate and 3 g l^−1^ gelrite) significantly improved hazel callus growth. This modified MS medium increased callus fresh weight (55.8%) as compared to the control. M_10_ medium increased fatty acids yield of callus (66.7%) as compared to the control. Liquid M_10_ medium maintained growth over a longer period of time and also increased slightly, the paclitaxel production as compared to the control. This novel medium is promising for facilitating the mass production of hazel callus as a source of valuable metabolites including paclitaxel, linoleic and oleic acids.

## Introduction

Paclitaxel is a powerful antimitotic agent with excellent activity against a range of cancers^1^. The major limitation in the extensive use of this valuable secondary metabolite is its low supply, since *Taxus* spp. contains very low amounts of paclitaxel^2^. Extraction of paclitaxel from this tree has imposed important ecological effects, resulting in the extinction of *Taxus* species^3^. Plant cell suspension culture is considered as the most promising approach to the production of paclitaxel^4^. The availability of this drug is still restricted and its cost is very high, mainly due to the recalcitrant behavior of *Taxus* spp. under *in vitro* conditions^2^. Therefore, the search for alternative sources of paclitaxel was considered as crucial. In addition to *Taxus* spp., hazel (*Corylus avellana)* has also been described as a paclitaxel-producing species through bioprospection among angiosperms^5,6^. The major advantages of producing paclitaxel through hazel cell cultures are that hazel is widely accessible and its *in vitro* cultivation is easier than that of yew^2,7^. It is stated that *in vitro* cultures of *C*. *avellana* can become a promising and cheaper source for paclitaxel production^8^. Besides the use of the nuts of hazel tree as a source of protein, its leaves are used to relieve the symptoms of hemorrhoidal and varicose veins^9^. The kernel and green leaf/flower portions of the hazel tree have antioxidant activity^10^. It is found that the consumption of nuts is protective against cardiac morbidity and mortality^11^.

In addition to the use of hazel cell cultures for paclitaxel production, hazel plantlets can be regenerated from callus tissues by differentiation induced by exogenous growth regulators. Plant regeneration from calli is possible by somatic embryogenesis or *in vitro* organogenesis. Meanwhile, infrequent somaclonal variants resulting from genetic diversity in somatic cells, mutations, chromosome aberrations and environmentally induced epigenetic changes can be isolated by plant regeneration of callus^12^.

Fast-growing callus is a key prerequisite for the success of mass callus production and then paclitaxel production, plant regeneration and transformation. Therefore, optimizing the culture medium for improvement of callus growth is a crucial step in mass callus production. For study of *in vitro* production of metabolites, in addition to a suitable protocol for callus induction, obtaining large amounts of callus biomass is a prerequisite^13^. Also, setup of fast-growing *in vitro* cultures is an important stage for producing secondary metabolites from the plant cell cultures^14^. One of the key problems in commercial production of secondary metabolites by plant cell cultures is slow growth of plant cells. The large-scale culture of low-growing cells is expensive and also basic laboratory analysis of such cells is tedious and difficult. Additionally, such slow growth rates require extreme precautions against contamination^15^.

Regulation of medium nutrients significantly improves the callus growth. There are a few reports on the influence of medium composition on embryogenic induction^16^ and also, the best method for inducing shoot organogenesis^17^ in hazel, but no sufficient information is available regarding the influence of different nutrient concentrations in the culture medium on callus growth of *C*. *avellana*. Recently, a suitable callus was obtained from *C*. *avellana* in our laboratory and optimization of hazel callus mass production was tried. For this purpose, effects of different concentrations of some inorganic ingredients of Murashige and Skoog (1962)^18^ (MS) as well as the effects of spirulina powder (*Arthrospira platensis*), casein hydrolysate and some amino acids on hazel callus production were investigated.

## Results and Discussion

The results of different MS medium amendments for improvement of growth of hazel callus indicated that these amendments enhanced callus growth.

### Effects of gelrite, pH 6.0 and medium volume increment on hazel callus growth

The 22 modifications (Table S1) in the first experiment significantly affected all the studied characteristics (Table S2). Accordingly, medium volume increment resulted in the highest amount of relative growth rates (RGR) (0.069 d^−1^) and relative fresh weight growth (RFWG) (4.65) which was significantly higher than that in the control (0.054 d^−1^and 2.87, respectively). In the present study, improved RGR and RFWG were obtained by the use of 3 g l^−1^ gelrite as the gelling agent (Table S2). Bacterial gellan gum (like gelrite^TM^ and phytagel^TM^) is the superior gelling agent for most plant tissue culture media due to its consistent quality and high purity^19^. It was reported that gelrite is the best gelling agent for callus growth in *Ilex paraguariensis* ^20^. The presence of ionic impurities in both gelrite and agar may affect growth^21,22^. Agar contains a large amount of sodium and also levels of sulfur and copper are significant. Gelrite has less organic impurities but inorganic ones exist at high concentrations^23,24^. The addition of 4 g l^−1^ gelrite as the gelling agent decreased significantly, RGR and RFWG in comparison with 3 g l^−1^ gelrite (Table S2). Indeed, as a result of the rigidity of the gelling agents, water and nutrient uptake was reduced. Therefore, the growth of callus was reduced. It was reported that gelling agent type and its concentration affect water availability and cytokinin uptake^21,22^. Furthermore, the chemical and physical characteristics of a culture medium are influenced by both the brand and concentration of the gelling agent^22^.

Increasing the amount of myoinositol to 3 times as compared to the control resulted in the lowest RGR and RFWG with an average of 0.039 d^−1^ and 1.64, respectively. Subsequently, omission of plant hormones in the medium caused lowest RGR and RFWG with an average of 0.041 d^−1^ and 1.80, respectively (Table S2). Improving callus growth with increasing amount of myoinositol up to an optimum concentration and decrease in callus growth with concentration beyond the optimum level in Vitis has been reported^25^. No significant difference in callus growth was observed between the control and T_22_ (supplemented with 2 mg l^−1^ GA_3_) (Table S2). While auxins^26^ and cytokinins^27^ are required for the growth of tissue cultures, the need for gibberellic acid is controversial^28^.

Since pH of the medium influences the uptake of nutrients by regulating their solubility, the adjustment of the medium pH is necessary^29^. According to the obtained results, MS medium with pH 6.0 is recommended for the production of hazel callus (Table S2). Effect of the medium pH on callus growth of *Aquilaria malaccensisis* was reported^30^. No significant difference in hazel callus growth was obtained by replacement of FeEDTA by FeEDDHA, nor by increasing the amount of KNO_3_ up to 1.5 times and also using half the amount of NH_4_NO_3_ or KNO_3_, Whereas simultaneous decrease in KNO_3_ and NH_4_NO_3_ by half, reduced significantly, callus growth (Table S2). Nitrogen plays a major role in growth. The cell growth is affected by the form and amount of nitrogen source in culture medium^31^. Maintaining cultured cells in an undifferentiated state requires an easily attainable supply of nitrogen^32^. Doubled amount of NH_4_NO_3_ as compared to the control reduced callus growth (Table S2). This could be due to the toxicity of the ammonium ion at high concentration.

### Effect of casein hydrolysate on growth of hazel callus

Adding six concentrations of casein hydrolysate (0, 500, 1000, 1500, 2000, 2500 and 3000 mgl^−1^) to MS medium increased the weight and growth rate of hazel callus (Fig. S1). The highest RGR and RFWG of callus with an average of 0.068 d^−1^and 4.44, respectively were obtained by using 1000 mgl^−1^ of casein hydrolysate in MS medium which was significantly higher than that of the control. It was reported that media supplemented with casein hydrolysate can improve callus growth in different plants^33,34^. Casein hydrolysates contain calcium, phosphate, microelements, vitamins and up to 18 amino acids. Among commercially available casein hydrolysates, the supplies provided by enzymatic hydrolysis are favorable. There is a limit to the amount of casein hydrolysate which can be safely used in the culture medium. It is remarked that casein hydrolysate promotes growth in cultures where phosphate deficiency inhibit growth, suggesting that this deficiency is compensated for, by amino acids. It has been inferred that casein hydrolysate is also a source of phosphate^35^.

### Effect of spirulina powder on growth of hazel callus

*Arthrospira platensis*, also known as spirulina, is a multicellular and filamentous blue-green microalga. It is an edible microbe with a high food value and provides high levels of vitamins, minerals, β-carotene, essential fatty acids and antioxidants^36^. This investigation on hazel explores the effect of spirulina powder on callus growth rate. Calli were cultured on MS medium supplemented with different concentrations of spirulina powder (0, 100, 500, 1000, 1500 and 2000 mg l^−1^). The results indicated that spirulina powder in the medium significantly affected hazel callus growth. The highest RGR and RFWG of callus with an average of 0.072 d^−1^ and 5.03, respectively, were obtained by using 1000 mg l^−1^ of spirulina powder in MS medium, which was significantly higher than that of the control with a mean of 0.056 d^−1^ and 3.10, respectively (Fig. S2). The favorable consequence of seaweed extract on growth, yield, quality and environmental stress tolerance of crops has been already shown in *in vivo* conditions^37,38^. Acadian marine plant extract powder (AMPEP) is another alga powder that can improve callus growth. This alga powder is obtained from fresh *Ascophyllum nodosum* and contains the major and minor nutrients, carbohydrates, amino acids and plant growth promoting substances which are required for callus growth^39^.

### Effects of amino acids supplementation on growth of hazel callus

The present investigation showed the effects of some amino acids (glutamine, proline, alanine, phenylalanine, cysteine and methionine) at different concentrations (0, 50, 100, 150 and 200 mgl^−1^) on the growth of hazel callus. As shown in Fig. S3, the supplementation of these amino acids in culture media resulted in additive effects on RGR and RFWG of callus. Results indicated that using MS medium supplemented with any studied concentration of glutamine, alanine or methionine (Fig. S3) improved callus growth, but the maximum RGR and RFWG of callus were obtained by the use of 50 mg l^−1^ of glutamine, alanine or methionine. MS medium supplemented with any concentration of proline, phenylalanine or cysteine improved callus growth (Fig. S3). No significant difference was observed between different concentrations of proline, phenylalanine or cysteine (Fig. S3). Therefore, the addition of 50 mg l^−1^ proline, phenylalanine and cysteine to medium is recommended. Increased rate of callus growth by amino acid supplements have been reported^40,41^. Amino acids are an accessible nitrogen source for plant cells and can be absorbed much more readily than inorganic in the same medium^42^. According to the results of some studies^43,44^, amino acids are not necessary ingredient for many cultural purposes but their addition to the medium can compensate for medium deficiency or provide an accessible source of nitrogen to cultured cells or tissues. With ammonium ion uptake, plant tissues use adenosine triphosphate (ATP) as an energy source to convert it into amino acids^45^. Therefore, the presence of suitable amino acids in the medium may save some ATPs.

### Optimized culture medium for callus growth of hazel

According to the results obtained in the preliminary experiments, some treatments including 3 g l^−1^ gelrite as the gelling agent, medium pH 6.0, the use of 70 ml of medium instead of 50 ml and the addition of 1000 mgl^−1^ of casein hydrolysate, 1000 mgl^−1^ of spirulina powder, 50 mgl^−1^ of glutamine, proline, alanine, phenylalanine, cysteine and methionine, improved hazel callus growth. Thus, in order to find the optimal culture medium for improvement of hazel callus growth, 14 new modified MS media were prepared and investigated in another study (Table 1). In this final experiment, five grams of callus were cultured in each replication and the data was analyzed after 35 days.

**Table 1:** Different modified media (based on MS medium) tested for improving hazel callus growth.

Treatment	Medium pH = 6.0	Phytagel (3 g l^−1^)	Spirulina powder (1000 g l^−1^)	Glutamine (50 mg l^−1^)	Proline (50 mg l^−1^)	Alanine (50 mg l^−1^)	Phenylalanine (50 mg l^−1^)	Cysteine (50 mg l^−1^)	Methionine (50 mg l^−1^)	casein hydrolysate (1000 g l^−1^)	Glycine (50 mg l^−l^)	FeEDDHA (96 mg l^−1)^
M_0_	—	—	—	—	—	—	—	—	—	—	—	—
M_1_	*	*	—	—	—	—	—	—	—	—	—	—
M_2_	*	*	*	—	—	—	—	—	—	—	—	—
M_3_	*	*	*	*	—	—	—	—	—	—	—	—
M_4_	*	*	*	*	*	—	—	—	—	—	—	—
M_5_	*	*	*	*	*	*	—	—	—	—	—	—
M_6_	*	*	*	*	*	*	*	—	—	—	—	—
M_7_	*	*	*	*	*	*	*	*	—	—	—	—
M_8_	*	*	*	*	*	*	*	*	*	—	—	—
M_9_	*	*	*	*	*	*	*	*	*	*	—	—
M_10_	*	*	*	—	—	—	—	—	—	*	—	—
M_11_	*	*	*	—	—	—	—	—	—	*	*	—
M_12_	*	*	*	—	—	—	—	—	—	*	—	*
M_13_	*	*	*	*	*	*	*	*	*	*	—	*
M_14_	*	*	*	—	—	—	—	—	—	*	*	*

Volume of medium in all treatments was 70 ml.

**Figure 1:**
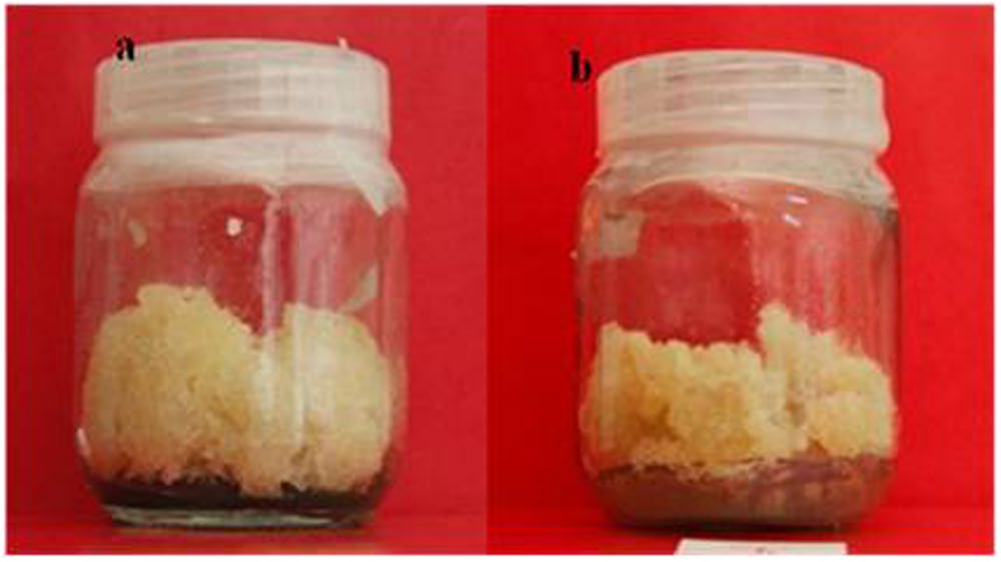
Hazel callus in M_10_ (**a**) and control (**b**) media.

The results presented in Table 2, clearly show that the new modified MS medium, M_10_, resulted in the highest amount of fresh weight (63.06 g), dry weight (1.70 g), RGR (0.072 d^−1^) and RFWG (11.61) which were significantly higher than that in the control (with a mean of 40.48 g, 1.06 g, 0.0597 d^−1^ and 7.10, respectively) (Fig. 1). The M_10_ medium contained 70 ml of culture medium per replication with pH 6.0 and supplemented with 1000 mg l^−1^ spirulina powder, 1000 mg l^−1^ casein hydrolysate and 3 g l^−1^ gelrite. It is noteworthy that the difference between M_9_ (M_10_ medium supplemented with 50 mg l^−1^ of six above-mentioned amino acids), M_10_ and M_11_ (M_10_ medium supplemented with 50 mg l^−1^ of glycine) was not significant (Table 2). Since M_10_ medium is less costly than M_9_ and M_11_, it is economically preferable. Therefore, using the M_10_ medium to enhance callus growth of *C*. *avellana* is advised. M_10_ medium (M_2_ medium supplemented with 1000 mg l^−1^ casein hydrolysate) resulted in a significantly higher RGR and RFWG than the M_8_ medium (M_2_ medium supplemented with 50 mg l^−1^ of six above-mentioned amino acids) (Table 2). Indeed, casein hydrolysate was more effective for hazel callus growth than the addition of the major amino acids. It is thought that casein hydrolysate might contain some unknown growth promoting factors. Data shown in Table 2 indicated that M_12_ medium (M_10_ medium supplemented with FeEDDHA) reduced callus growth as compared to M_10_ medium. As shown in Fig. S4, culture medium was not consumed by the hazel callus. It seems that the absorption and transport of nutrient elements are impaired in this modified MS medium.

**Table 2:** Effects of different modified MS media on fresh weight (FW), dry weight (DW), relative growth rate (RGR), relative fresh weight (RFWG) and percentage of callus water content of hazel callus (PCWC).

Treatment	FW	DW	RGR	RFWG	PCWC
M_0_	40.48g	1.06e	0.0597g	7.10g	97.37a
M_1_	51.46e	1.33d	0.0666e	9.29e	97.42a
M_2_	56.58d	1.47c	0.0693d	10.32d	97.41a
M_3_	58.48c	1.53bc	0.0702cd	10.70c	97.38a
M_4_	59.06bc	1.54bc	0.0705bc	10.81bc	97.40a
M_5_	59.18bc	1.53bc	0.0706bc	10.84bc	97.41a
M_6_	59.20bc	1.55b	0.0706bc	10.84bc	97.39a
M_7_	59.33bc	1.55b	0.0707bc	10.87bc	97.38a
M_8_	60.73b	1.58b	0.0713b	11.15b	97.40a
M_9_	64.07a	1.68a	0.0729a	11.81a	97.38a
M_10_	63.06a	1.70a	0.0724a	11.61a	97.30a
M_11_	63.77a	1.67a	0.0727a	11.75a	97.38a
M_12_	34.38h	1.12e	0.0551h	5.88h	96.73b
M_13_	41.20fg	1.32d	0.0602fg	7.24fg	96.79b
M_14_	41.20fg	1.32d	0.0602fg	7.24fg	96.79b
LSD (0.05)	1.78	0.07	0.0011	0.36	0.14

Means within a column followed by the same letter are not significantly different (p ≤ 0.05).

### Effect of optimized M_10_ medium on fatty acids profile of hazel callus

**Figure 2:**
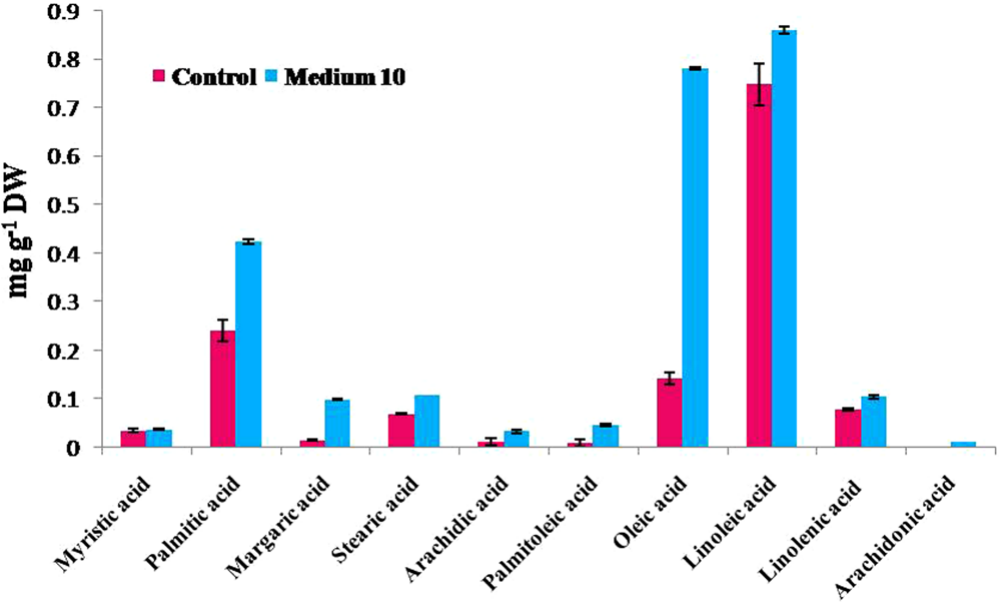
Effects of control and M_10_ medium on the fatty acids of hazel callus.

The seed oil of *C*. *avellana* and *C*. *americana* can be served as occlusive skin-conditioning agents^46^. Nut oil of *C*. *avellana* was reported as a natural cosmetic hazel oil without contaminants, which is used in 85 cosmetic formulations^46^. Analysis of *C*. *avellana* fatty acids profile indicated that linoleic acid (C18:2) in M_0_ medium and the linoleic acid (C18:2) and oleic acid (C18:1) in M_10_ medium were the main fatty acids in the fatty acids profile (Fig. 2). The proportion of unsaturated fatty acids was higher than the saturated acids. Palmitic acid (C16:0) and linoleic acid (C18:2) were the major components of saturated and unsaturated fatty acids, respectively. Short (myristic acid, C14:0) and long chain fatty acids (arachidic acid, C20:0) were detected in small amounts. Arachidonic acid (C20:4) was detected only in M_10_. Unsaturated, saturated and total fatty acids of callus in M_10_ were higher than in M_0_ according to Student’s t-test. It is reported that spirulina (*Arthrospira platensis*) is the source of essential fatty acids^47^. High fatty acid content of M_10_ as compared to the control can be explained by the amendments used in M_10_ medium including spirulina powder as a source of essential fatty acids while the control was spirulina powder-free medium. Linoleic and oleic acids which are used in cosmetic and pharmaceutical products^48^ represent 91% of the total lipids of callus grown in M_10_. Previous report^49^ showed that α-linolenic acid and its ester derivatives have strong antimicrobial activity against various oral pathogens.

### Effect of optimized M_10_ medium on cell growth and paclitaxel production in hazel cell suspension culture

**Figure 3:**
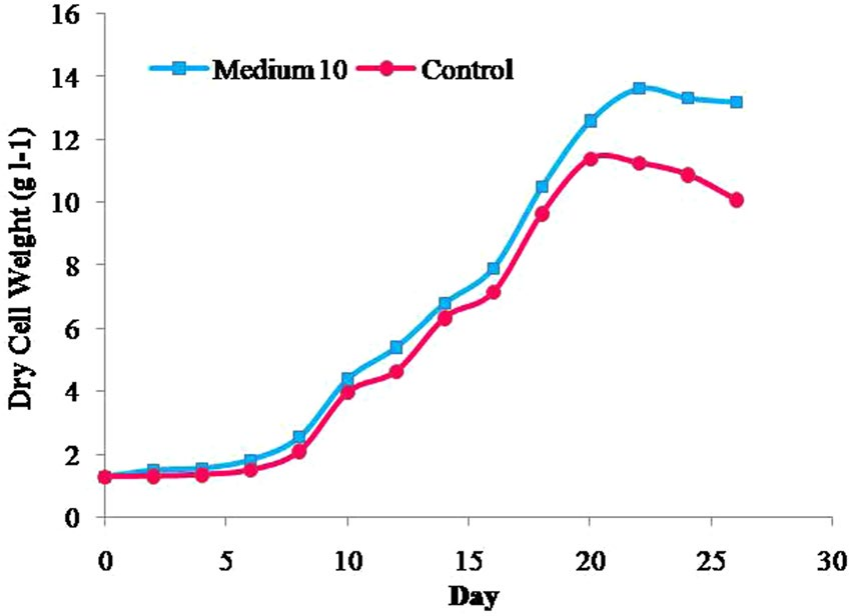
Time courses of hazel cell growth in control and in M_10 _medium. Average values of three replicates are given.

Growth trends of the hazel cell suspensions showed that cell growth in M_10_ reached the stationary phase 2 days later as compared to the control (Fig. 3). The maximum dry weight in the control and M_10_ were about 11.39 and 13.64 g l^−1^, respectively (Fig. 3). The average growth rate over the growth period (AGR = [maximum cell density − initial cell density]/growth period^50^) was about 0.50 and 0.56 g l^−1^ day^−1^ in the control and M_10_, respectively. The cell growth index (maximum cell density/initial cell density^50^) in the control and M_10_ were about 8.78 and 10.49, respectively. The maximum biomass in M_10_ was 19.8% greater than that in the control. It was considered that this higher biomass was achieved by higher average growth rate and also by maintaining growth over a longer period of time (Fig. 3).

**Figure 4:**
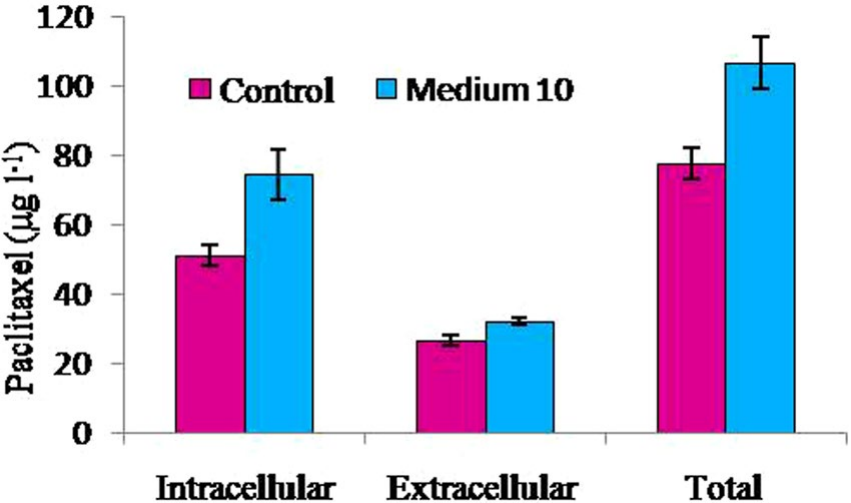
Effects of control and M_10_ medium on paclitaxel production (**a**) and dry weight (**b**).

The average growth rate of M_10_ was higher than that of the control and the hazel cells grown in M_10_ medium were able to maintain positive growth during a period of 22 days, whereas the cells grown in the control reached stationary phase within 21 days. These trends may be explained by the amendments used for M_10_ medium including major and minor nutrients, carbohydrates, amino acids and plant growth promoting substances present in spirulina powder and casein hydrolysate that were absent in the control. The maximum total yield of paclitaxel concentration in the control (77.7 µg l^−1^) and M_10_ medium (106.6 µg l^−1^) was obtained on days 21 and 23, respectively and was produced mainly in the cells (Fig. 4). The dry weight of cells and the intracellular, extracellular and total yield of paclitaxel increased significantly by using M_10_ medium according to Student’s t-test (Table S3).

## Conclusion

Paclitaxel production by *C*. *avellana* cell culture was found for the first time in 2006^2^ and the use of hazel cell culture for paclitaxel production is promising. Establishment of fast-growing *in vitro* cultures by using the most suitable medium is a key step towards producing paclitaxel from the hazel cell cultures. However, there are no considerable reports regarding the influences of different nutrient concentrations in the culture medium on callus growth of *C*. *avellana*. Therefore, the effects of different concentrations of mineral elements, spirulina powder, casein hydrolysate and some amino acids in MS medium on improvement of the production of hazel callus, was investigated. The modified MS medium (pH 6.0) with 1000 mg l^−1^ algae powder, 1000 mg l^−1^ casein hydrolysate and 3 g l^−1^ gelrite favored the increase of callus biomass of *C*. *avellana*. M_10_ medium as the best treatment increased the fatty acids yield of hazel callus (66.7%) as compared to the control. Also, M_10_ liquid medium increased the culture period and cell biomass in cell suspension culture. Production of paclitaxel by hazel cell suspension was not as high as that reported for *Taxus* cell suspension. However, it should be noted that establishment of the fast-growing *in vitro* culture of hazel may compensate for the lower yield of paclitaxel. It is noteworthy that paclitaxel production in this cell clone was improved by the combined use of phenylalanine and Vanadyl sulfate^51^. Therefore, it is recommended to improve paclitaxel productivity in two stage cell culture system. The use of M_10_ liquid medium may favour increase of cell population by improving growth at the first stage of culture (logarithmic phase). Besides, the use of different elicitors and precursors at the second stage (stationary phase) can lead to increased production of paclitaxel via involvement of some related pathways. Also, the lipids of hazel callus may have large clinical and economic applications due to their content of unsaturated fatty acids (linoleic and oleic acids) that are used in cosmetic and pharmaceutical products^48^. The cell suspension of *C*. *avellana* as a promising source of paclitaxel can be effective in reducing the high cost of drug-therapy.

## Materials and Methods

### Plant materials and tissue culture reagents

The *C*. *avellana* callus was obtained from a stable 6-year-old diploid callus. Concisely, callus were derived from seed cotyledons on MS medium supplemented with 0.2 mg l^−1^ 6-benzylaminopurine and 2 mg l^−1^ dichlorophenoxyacetic acid, and solidified with 8 g l^−1^ agar agar. The pH of all media was adjusted to 5.8 with either KOH or HCl prior to autoclaving for 20 min at 121 °C. All cultures were incubated in dark at 25 ± 2 °C until the calli emerged. These calli were routinely subcultured every 25 days. To obtain a homogenous callus, several subcultures of calli were carried out on the same medium.

The medium components, plant growth regulators, paclitaxel and fatty acids standards used in the experiments were supplied by Sigma and Merck Chemical Companies.

### Culture amendment experiments

Nine independent experiments with five replications were carried out in this research. All experiments except the first one were planned based on Completely Randomized Design (CRD), and the first experiment was set up in a Randomized Complete Block Design (RCBD). In all experiments, each replication consisted of a glass jar with autoclave-resistant plastic caps (5.5 cm in diameter, 8 cm in height and 250 ml in volume) containing 50 ml medium and seven grams of hazel callus. The cultures were incubated in a controlled incubator at 25 °C for 25 days in the dark. The first experiment was set up to test effects of 22 modifications applied in MS medium (Table S1) for improvement of hazel callus growth.

The second experiment was performed to assess the effects of different concentrations of casein hydrolysate (0, 500, 1000, 1500, 2000, 2500 and 3000 mg l^−1^) on callus growth of *C*. *avellana*. The third experiment was designed to study the effect of spirulina (*Arthrospira platensis*) powder in medium (0, 100, 500, 1000, 1500 and 2000 mg l^−1^) on the fresh and dry weights of hazel calli. The next six independent experiments were planned to evaluate separately, the effects of different levels (0, 50, 100, 150 and 200 mg l^−1^) of six amino acids (glutamine, proline, alanine, phenylalanine, cysteine and methionine) on callus growth of *C*. *avellana*.

### Growth indices and water content of callus

In all the experiments, two growth indices and water content of callus were investigated as follows:

*Relative growth rate* (*day* ^*−1*^). Calli were weighted before culturing on callus production medium and were weighted once again 25 days after culture (W1). Relative growth rate (RGR) was calculated based on fresh weight according to Eq. (1)^52^.

1 $${\rm{RGR}}({{\rm{d}}}^{-1})=[\mathrm{ln}({{\rm{W}}}_{{\rm{1}}})-\,\mathrm{ln}({{\rm{W}}}_{{\rm{0}}})]/{\rm{growth}}\,{\rm{period}}$$

*Relative fresh weight growth (RFWG)*. Relative fresh weight growth of callus was calculated according to Eq. (2).

2 $${\rm{RFWG}}=[({\rm{W}}1-W0)]/W0$$

*Percentage of callus water content (PCWC)*. All samples of calli were dried to constant weight at 60 °C for 36 h in an oven. This trait was calculated according to Eq. (3).

3 $${\rm{PCWC}}=[({\rm{Freshweight}}\,-\,{\rm{Dryweight}})/{\rm{Freshweight}}]\times 100$$

### Lipid analysis

Calli were dried in the oven at 60 °C. Lipid extraction, preparation of the fatty acid methyl esters and GC/MS analysis for *C*. *avellana* callus was done according to the procedure described by Bao *et al*.^53^. All samples were filtered through 0.22 µm cellulose acetate syringe filters before analysis with GC/MS. The fatty acids in samples were analyzed by Hewlett Packard 5890 gas chromatograph MSD 5972 mass analyzer with a HP-5MS capillary column (Agilent Technologies, Santa Clara, CA).

### Measurement of cell growth

The *C*. *avellana* cell suspension cultures were obtained by cultivating 5 g callus into 250 ml Erlenmeyer flask containing 100 ml of MS medium supplemented with 0.2 mg l^−1^ BAP and 2 mg l^−1^ 2,4-D acid and maintained at 25 °C in darkness on gyratory shakers at 110 rpm. Cell suspensions were also subcultured every 15 days until the cells reached homogeneity. Then, 1.5 ± 0.1 g of cells (fresh mass) were cultivated in 100 ml Erlenmeyer flask containing 30 ml MS medium.

The cell growth was determined by measuring the dry cell weight (DCW). Briefly, the biomass in the cell suspension culture was separated from the liquid medium by filtration (Whatman No. 1) and then dried at 60 °C to constant weight to obtain the dry cell weight.

### Quantification of paclitaxel

Hazel cells were separated from cell suspension culture through a filter paper (Whatman No. 1). The cell-free medium was subsequently extracted according to the method proposed by Fett-Neto^54^. Intracellular paclitaxel was extracted from the cells with a procedure described by Luo *et al*.^55^. All samples were filtered through 0.22 µm cellulose acetate syringe filters before analysis with HPLC. Paclitaxel in samples was analyzed by HPLC (Waters, USA) with a C18 analysis column (MachereyeNagel EC 250/4.6 Nucleodur). The sample (20 µl) was injected each time and detected at 230 nm using a UV detector. The mobile phase was methanol: water (80:20 v/v) at a flow rate of 1.0 ml/min. The quantification of paclitaxel was based on an external standard of genuine paclitaxel (Sigma).

### Statistical analysis

The hypothesis of normality and equal variance were met and conventional parametric statistics was used for the analysis. Analysis of variance and means comparison using least significant difference (LSD) were performed by SAS (SAS 9.1, 2003) and Excel (Excel, 2013) software was used for making graphs.

### Availability of data and material

The dataset supporting the conclusions of this article is included in the article.

## Electronic supplementary material

Supplementary Information
