# Supplementary material for: A Novel Medium for Enhancing Callus Growth of Hazel (Corylus avellana L.)
Source: Sci Rep. 2017 Nov 15;7:15598. doi: 10.1038/s41598-017-15703-z (PMC5688170; doi:10.1038/s41598-017-15703-z)
Supplement: Supplementary file 1 — Supplementary Information [file 41598_2017_15703_MOESM1_ESM.doc]

## **Supplementary Information**

## **A Novel Medium for Enhancing Callus Growth of Hazel (*Corylus avellana* L.)**

Mina Salehi^1^, Ahmad Moieni^1*^, Naser Safaie^2^

^1^ plant Breeding and Biotechnology Department, Faculty of Agriculture, Tarbiat Modares University, Tehran, P.O. Box 14115-336, Iran

^2^ Plant Pathology Department, Faculty of Agriculture, Tarbiat Modares University, Tehran, P.O. Box 14115-336, Iran

*Corresponding author: moieni_a@modares.ac.ir

1. **Figure Legends**

**Figure S1.** Effects of different concentrations of casein hydrolysate on fresh weight (FW), dry weight (DW), relative growth rate (RGR) and relative fresh weight (RFWG) of hazel callus. Average values are given, error bars are represented by vertical lines.

**Figure S2.** Effects of different concentrations of spirulina powder on fresh weight (FW), dry weight (DW), relative growth rate (RGR) and relative fresh weight (RFWG) of hazel callus. Average values are given, error bars are represented by vertical lines.

**Figure S3.** Effects of different concentrations of glutamine, proline, alanine, phenylalanine, cysteine and methionine on fresh weight (FW), dry weight (DW), relative growth rate (RGR) and relative fresh weight (RFWG) of hazel callus. Average values are given, error bars are represented by vertical lines.

**Figure S4.** Hazel callus in MS medium; M_10_ (a) and M_12_ (b)


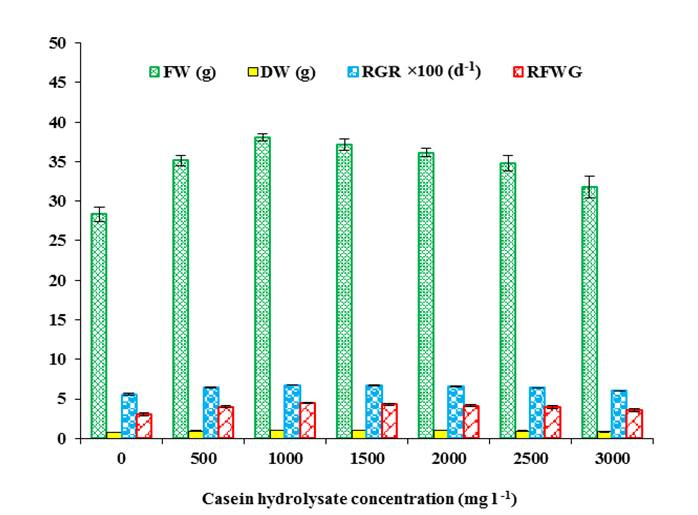


**Figure S1**


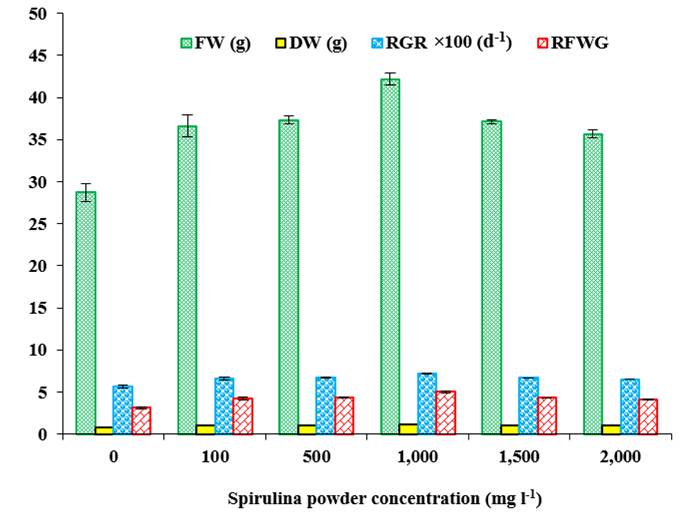


**Figure S2**


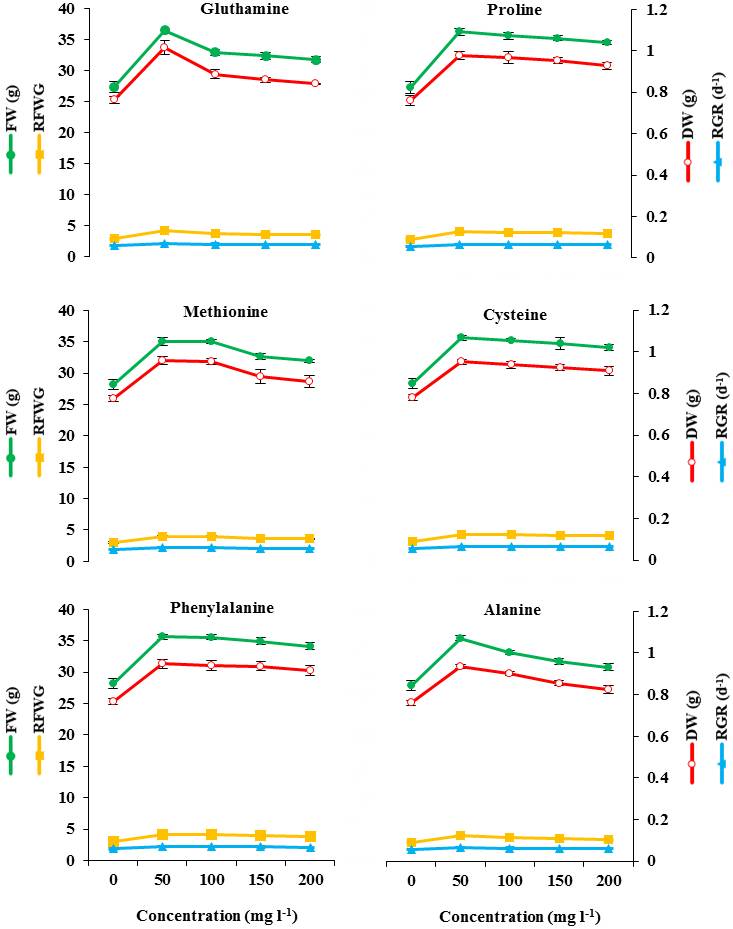


**Figure S3**


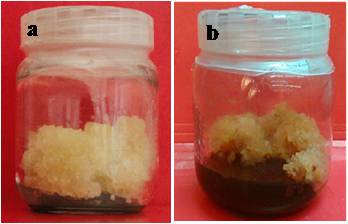


**Figure S4**

1. **Table Legends**

**Table S1.** Treatments tested for improvement of hazel callus growth.

**Table S2.** Effects of different treatments on the fresh weight (FW), dry weight (DW), relative growth rate (RGR), relative fresh weight (RFWG) and the percentage of callus water content (PCWC) of hazel callus.

**Table S3.** Results of t-test and descriptive statistics for equality of mean.

| **Table S1.** Treatments tested for improvement of hazel callus growth. | |
| --- | --- |
| Treatments | Definition |
|  |  |
| T_1_ | Half amount of KNO_3_ and NH_4_NO_3_ compared to control medium (T_10_) |
| T_2_ | Adding 3 g l^-1^ gelrite as the gelling agent |
| T_3_ | Adding 4 g l^-1^ gelrite as the gelling agent |
| T_4_ | Medium pH=6.0 |
| T_5_ | Medium pH=5.6 |
| T_6_ | Double amount of NH_4_NO_3_ compared to control medium (T_10_) |
| T_7_ | Half-strength macroelement compounds compared to control medium (T_10_) |
| T_8_ | Using 96 mg l^-1^ of FeEDDHA (ferric sodium complex and Ethylene diamine-N,N′-bis (2-hydroxyphenylacetic acid) |
| T_9_ | Control medium^*^ |
| T_10_ | Increasing the volume of medium (70 ml) per 250 ml culture vessel |
| T_11_ | Using half amount of NH_4_NO_3_ than the control |
| T_12_ | Using half amount of KNO_3_ than the control |
| T_13_ | Increasing the amount of KNO_3_ to 1.5 times more than control |
| T_14_ | Increasing the amount of MgSO_4_ to 1.5 times more than control |
| T_15_ | Increasing the amount of CaCl_2_ to 1.5 times more than control |
| T_16_ | Increasing the amount of ZnSO_4_ to 1.5 times more than control |
| T_17_ | 20 % increase in iron (FeEDDHA) than the T_8_ medium |
| T_18_ | Control medium but without hormones |
| T_19_ | Increasing the amount of NH_4_NO_3_ to 1.5 times, increasing the amount of CaCl_2_ 1.5 times, 20 % increase in iron supplementation (FeEDDHA), 6 g l ^-1^ agar-agar and 2 g l^-1^ gelrite, increasing the volume of medium (70 ml) per 250 ml culture vessel |
| T_20_ | Increasing the amount of myoinositol to 2 times more than control |
| T_21_ | Increasing the amount of myoinositol to 3 times more than control |
| T_22_ | Control medium but with 2 mg l^-1^ Gibberellic acid (GA_3_) |
|  |  |
|  | |
| ^*^Control medium: NH_4_NO_3_ 1.650 mg l^-1^, KNO_3_ 1.900 mg l^-1^, CaCl_2_·2H_2_O 440 mg l^-1^, MgSO_4_·7H_2_O 370 mg l^-1^, KH_2_PO_4_ 170 mg l^-1^, Na_2_–EDTA 37.25 mg l^-1^, FeSO_4_·7H_2_O 27.85 mg l^-1^, MnSO_4_·4H_2_O 22.3 mg l^-1^, ZnSO_4_·4H_2_O 8.6 mg l^-1^, H_3_BO_3_ 6.2 mg l^-1^, KI 0.83 mg l^-1^, Na_2_MoO_4_·2H_2_O 0.25 mg l^-1^, CuSO_4_·5H_2_O 0.025 mg l^-1^, CoCl_2_·6H_2_O 0.025 mg l^-1^, glycine 2 mg l^-1^, nicotinic acid 0.5 mg l^-1^, pyridoxine–HCl 0.5 mg l^-1^, thiamine–HCl 0.1 mg l^-1^, myoinositol 100 mg l^-1^, agar-agar 8 g l^-1^, BAP 0.4 mg l^-1^, 2,4-D 4 mg l^-1^; volume of medium was 50 ml. | |

| **Table S2.** Effects of different treatments on the fresh weight (FW), dry weight (DW), relative growth rate (RGR), relative fresh weight (RFWG) and the percentage of callus water content (PCWC) of hazel callus. | | | | | |
| --- | --- | --- | --- | --- | --- |
| Treatments | FW | DW | RGR | RFWG | PCWC |
|  |  |  |  |  |  |
| T_1_ | 25fgh | 0.69ef | 0.051fgh | 2.57fgh | 97.23a |
| T_2_ | **33.47b** | **0.90b** | **0.062b** | **3.78b** | 97.31a |
| T_3_ | **29.32c** | **0.81c** | **0.057c** | **3.19c** | 97.24a |
| T_4_ | **34.01b** | **0.93b** | **0.063b** | **3.86b** | 97.27a |
| T_5_ | 27.04def | 0.74de | 0.054de | 2.86def | 97.28a |
| T_6_ | 24.32ghi | 0.68ef | 0.050ghi | 2.47ghi | 97.19a |
| T_7_ | 20.96kl | 0.59hi | 0.044kl | 1.99kl | 97.20a |
| T_8_ | 27.96cde | 0.75cde | 0.055cde | 3.00 cde | 97.31a |
| T_9_ | 27.12de | 0.74de | 0.054cde | 2.87de | 97.29a |
| T_10_ | **39.58a** | **1.06a** | **0.069a** | **4.65a** | 97.32a |
| T_11_ | 28.80cd | 0.77cd | 0.056cd | 3.11cd | 97.32a |
| T_12_ | 26.90def | 0.73de | 0.054def | 2.84def | 97.27a |
| T_13_ | 28.94cd | 0.78cd | 0.057cd | 3.13cd | 97.29a |
| T_14_ | 22.67ijk | 0.63fgh | 0.047ijk | 2.24ijk | 97.20a |
| T_15_ | 23.30hij | 0.65fgh | 0.048hij | 2.33hij | 97.23a |
| T_16_ | 21.56jkl | 0.60ghi | 0.045jk | 2.08jkl | 97.19a |
| T_17_ | 26.06efg | 0.79cd | 0.052efg | 2.72efg | 96.98b |
| T_18_ | 19.60lm | 0.55i | 0.041lm | 1.80lm | 97.19a |
| T_19_ | 24.12ghi | 0.66fg | 0.049ghi | 2.44ghi | 97.25a |
| T_20_ | 27.84cde | 0.76cd | 0.055cde | 2.98cde | 97.26a |
| T_21_ | 18.50m | 0.55i | 0.039m | 1.64m | 97.00b |
| T_22_ | 27.60cde | 0.74cde | 0.055cde | 2.94cde | 97.30a |
| LSD (0.05) | 2.12 | 0.07 | 0.003 | 0.30 | 0.13 |
| Means within a column followed by the same letter are not significantly different (*p*≤0.05). | | | | | |

| **Table S3.** Results of t-test and descriptive statistics for equality of mean. | | | | | | | | |
| --- | --- | --- | --- | --- | --- | --- | --- | --- |
| Traits | Treatment | | | | |  |  |  |
|  | M_0_ (Control) | |  | M_10_ | |  |  |  |
|  | Mean | n |  | Mean | n |  | t-test | df |
| Dry weight | 11.39 ± 0.17 | 3 |  | 13.64 ±0.18 | 2 |  | 8.9^**^ | 4 |
| Intracelluar paclitaxel (µg l^-1^) | 51.1 ± 2.9 | 3 |  | 74.37 ± 7.12 | 2 |  | 3.02 ^ns^ | 4 |
| Extracellular paclitaxel (µg l^-1^) | 26.6 ± 1.6 | 3 |  | 32.18 ± 0.94 | 2 |  | 3.01 ^*^ | 4 |
| Total paclitaxel (µg l^-1^) | 77.7 ± 4.46 | 3 |  | 106.6 ± 7.4 | 2 |  | 3.32^*^ | 4 |
